# Recall and recognition of in-game advertising: the role of game control

**DOI:** 10.3389/fpsyg.2013.01023

**Published:** 2014-01-24

**Authors:** Laura Herrewijn, Karolien Poels

**Affiliations:** Department of Communication Studies, University of AntwerpAntwerp, Belgium

**Keywords:** in-game advertising, game control, player involvement, brand prominence, brand awareness

## Abstract

Digital gaming has become one of the largest entertainment sectors worldwide, increasingly turning the medium into a promising vehicle for advertisers. As a result, the inclusion of advertising messages in digital games or in-game advertising (IGA) is expected to grow steadily over the course of the following years. However, much work is still needed to maximize the effectiveness of IGA. The aim of the study was to contribute to IGA effectiveness research by analyzing the impact of two factors on the processing of IGA in terms of brand awareness. The primary objective was to investigate the effect of a person’s sense of involvement related to the control and movement mechanisms in a game (i.e., kinesthetic involvement). A within-subjects experiment was conducted in which control over a racing game was varied by manipulating game controller type, resulting in two experimental conditions (symbolic versus mimetic controller). Results show that the variation in game controller has a significant effect on the recall and recognition of the brands integrated into the game, and that this effect can be partially brought back to players’ perceived control over the game: when a game is easier to control, the control mechanisms require less conscious attention, freeing attentional resources that can be subsequently spent on other elements of the game such as IGA. A second factor that was taken into account in the study was brand prominence. The influence of both the size and spatial position of in-game advertisements was examined. Findings demonstrate that there are significant changes in effectiveness between different types of placements. Spatial position seems to be the most important placement characteristic, with central brand placements obtaining the highest recall and recognition scores. The effect of ad size is much smaller, with the effectiveness of the large placements not differing significantly from the effectiveness of their smaller counterparts.

## INTRODUCTION

No other entertainment sector has experienced the same explosive growth as the digital game industry. A report from DFC Intelligence forecasts that the global market for digital games is expected to grow from $63 billion in 2012, to $78 billion in 2017 (DFC Intelligence, 2013), making it one of the largest entertainment sectors worldwide. Moreover, according to the Entertainment Software Association, 58% of U.S. citizens play digital games; 45% of all game players are women; and the average game player is 30 years old (32% of all game players are younger than 18 years, 32% are between 18 and 35 years, and 36% are over 36 years) and has been playing games for 13 years (Entertainment Software Association, 2013). These figures reflect the increasing popularity of digital games, showing that there are millions of people from all socio-demographic groups who increasingly enjoy playing digital games in their spare time. Digital games have thus surpassed their status as being a predominantly male pastime, and have grown into a mainstream entertainment medium that touches every segment of the population.

Consequently, the advertising industry has taken an interest in digital games. The appearance of advertising inside digital games goes as far back as the early 1970s, when the computer game *Lunar Lander*^1^ included a McDonald’s restaurant as a hidden feature or easter egg into its gameplay. The goal of *Lunar Lander* was to land a lunar module on the moon. If the player landed on exactly the right spot, the McDonald’s restaurant would appear and the astronaut would order a Big Mac hamburger to go. Crashing into the restaurant, however, destroyed it permanently and the game would display a message, scorning the player for destroying the only McDonald’s on the moon (Vedrashko, 2006; Skalski et al., 2010). In this early example of advertising inside a digital game, the brand was integrated because of its humoristic rather than commercial value. Advertisers began showing explicit interest in digital games in the early 1980s though, and from the 1990s on, advertisers began to see digital games as an appropriate and viable medium for the incorporation of their advertisements and the reaching of their target markets (Schneider and Cornwell, 2005; Vedrashko, 2006; Mau et al., 2008; Mackay et al., 2009; Nicovich, 2010; Skalski et al., 2010). This interest in the use of digital games as a medium for the delivery of advertisements has been increasing ever since, and is predicted to keep growing steadily over the next several years. On a global basis, advertising related to digital games is expected to reach $7.2 billion by 2016, up from $3.1 billion in 2010 (DFC Intelligence, 2011). This includes *in-game advertising* (IGA) or the incorporation of advertising messages into existing digital games, a practice similar to product placement in movies or television shows (Nelson et al., 2006; Bogost, 2007; Interactive Advertising Bureau, 2010). IGA comes in a lot of different formats, such as the inclusion of real-world analogs (e.g., billboards, poster ads, radio spots and television commercials), product placements, branded music, and branded characters in digital games. These formats have been maturing throughout the years, advancing from very static toward more dynamic types of IGA (Schneider and Cornwell, 2005; Vedrashko, 2006; Bogost, 2007; Bardzell et al., 2008). This implies that, due to the online capabilities of modern digital games, advertisements can now be dynamically delivered and updated in-game based on multiple criteria, such as players’ demographic, regional, and gamer profile, time of the day, etcetera (Schneider and Cornwell, 2005; Vedrashko, 2006; Bogost, 2007; Bardzell et al., 2008). Apart from reaching an ever-growing, diverse audience and the possibility to dynamically place, track, and alter ad units in games, the appeal of IGA also lies in the long shelf-life and replay value of games (the average game is played for up to 30 h), and the fact that integrating ads into digital games can provide brands with the opportunity to become an integral part of the digital game experience, reaching out to players in a highly vivid, interactive and immersive entertainment environment (Nelson, 2002, 2005; Schneider and Cornwell, 2005; Mackay et al., 2009; Nicovich, 2010). Moreover, for game publishers and developers, the integration of advertising is an interesting means to subsidize the rising development and marketing costs of their games without having to increase the retail price, which also benefits the gamer as end user (Chambers, 2005).

However, much work is still needed to maximize the effectiveness of IGA. Prior research has shown that IGA effectiveness often depends on a multitude of context-related factors, such as the type of brand or advertisement that is integrated, the prominence of the brand placement, the amount of congruence between game and product, the situational circumstances in which the ad is encountered, the emotions and experiences of the player during the encounter, etcetera (e.g., Nelson, 2002, 2005; Grigorovici and Constantin, 2004; Schneider and Cornwell, 2005; Mau et al., 2008; Mackay et al., 2009; Lewis and Porter, 2010). The aim of the current study is to further analyze the impact of this contextual component in an *experimental setting*, in order to come to a better understanding of the issues and mechanisms that are critical to the effective use of IGA.

As the starting point of our study, we take the *limited capacity model of motivated mediated message processing* [*LC4MP* (Lang, 2009)]. This model states that a person’s total attentional capacity (and thus his ability to cognitively process information) is limited. This has important implications for the effectiveness of IGA. Digital games are considered to be highly interactive and involving, with a multitude of tasks and stimuli vying for attention at the same time. Getting a brand noticed and remembered in such an involving game context is not self-evident, since people allocate their attentional resources to those aspects of an activity that are most relevant to them at a particular time. In a digital game context, this means that people will focus their attention primarily on the most essential tasks at hand, i.e., tasks and information that are central to furthering their progress in-game, while leaving fewer mental resources for the processing of secondary information such as advertisements that are embedded into the game (Grigorovici and Constantin, 2004; Lee and Faber, 2007; Lang, 2009). Keeping this in mind, there are several contextual factors that are important to consider when studying the effectiveness of IGA and the player’s ability to cognitively process these advertising messages. The current study specifically examines the impact of two of these factors on the processing of IGA in an experimental setting.

First of all, we look at the effect of a player’s sense of *involvement* in the game on the way he is able to process IGA. Games are an interactive, vivid, engaging, immersive, and complex cultural form that require an active audience (Nelson, 2005) and are able to induce a wide variety of emotions and experiences (Poels et al., 2012). These medium-specific characteristics are considered responsible for an enhanced level of audience (i.e., player) involvement (Vorderer, 2000). Prior research already showed that player involvement is a relevant factor to consider in an IGA context, showing that different levels of player involvement affect the way players process IGA in terms of brand awareness (i.e., brand recall and brand recognition; e.g., Grigorovici and Constantin, 2004; Lee and Faber, 2007).

However, involvement is a multidimensional construct and in the specific context of digital games, it is understood as a combination of six primary sources of engagement, namely: control and movement in the game environment (kinesthetic involvement); the exploration, navigation, and learning of the game’s spatial domain (spatial involvement); players’ awareness of and interaction with other agents in the game environment (shared involvement); the emotions that are generated during gameplay (affective involvement); story elements that have been written into a game, and those that emerge from the player’s interaction with the game (narrative involvement); and the pursuit of goals and the decision-making and reward systems integrated in a game (ludic involvement). These six dimensions occur with varying degrees of intensity and with frequent, fluid shifts in attention (Calleja, 2011).

This multidimensional nature of player involvement has not been taken into account in IGA effectiveness research before, however, implicating that the results of prior studies only lift a corner of the veil. Therefore, this study scrutinizes the effects of one dimension of player involvement, namely *kinesthetic involvement* or the player’s involvement related to the modes of control and movement in a game (Calleja, 2011), in order to be able to analyze how the attention toward and involvement with this specific component of the game influences how people process IGA. Player control and in-game movement are a central part of the digital gaming experience, creating a direct link between the player and his avatar in the game world that contributes to the interactive nature of games. There are a lot of different forms of game control, and the amount of freedom that is allowed and the nature and difficulty of the controls have a great impact on the player’s sense of involvement in the game environment (Calleja, 2011). The dimension requires more conscious attention when the player is still learning to use the game controls, or because a situation demands a complex sequence of actions that are very challenging (Calleja, 2011). The main objective of this study is to examine the impact of kinesthetic involvement on the processing of IGA in an experimental context by manipulating the *player’s control over the game world*.

Secondly, we investigate the impact of an additional contextual factor that might alter the processing of advertising inside a digital game environment, namely the *prominence* *of the brand placement*. Brand prominence is mostly defined as a factor that depends on placement characteristics such as ad size, color, attractiveness, and spatial position. Several IGA studies have already looked at the effect of these placement characteristics, showing that prominent brand placements are generally better in capturing the player’s attention, resulting in a positive effect on brand awareness (e.g., Grigorovici and Constantin, 2004; Schneider and Cornwell, 2005; Acar, 2007; Lee and Faber, 2007; Bardzell et al., 2008; Jeong and Biocca, 2012). However, these studies have focused on the impact of only one placement characteristic (i.e., ad size or spatial position) or on the influence of all characteristics at the same time. In the current study, we will elaborate on the effect of brand prominence by examining how both *ad size* and *spatial position* relate to people’s response to the brand placements, in different combinations.

## STUDY SET UP AND HYPOTHESES

### KINESTHETIC INVOLVEMENT

When playing a digital game, players have the opportunity to influence – to varying degrees – what happens in the game environment. The kinesthetic dimension of player involvement deals specifically with this exertion of agency, manifesting itself in the form of avatar control and the sensation of movement this can produce (Calleja, 2011). Consequently, kinesthetic involvement is closely connected to the modes of game control that are possible. Digital games can be controlled with a wide range of different input devices or game controllers that have progressed considerably over time. These modes of game control range from the more traditional, symbolic game controllers to the relatively new, motion-based symbiotic, and mimetic game controllers (Calleja, 2011; Skalski et al., 2011).

On one end of the spectrum, there is the symbolic control of controller buttons, keys, and thumb sticks, as used in the traditional keyboard and mouse combo and gamepads (e.g., traditional *Microsoft Xbox 360* and *Sony PlayStation 3* gamepad controllers). In the case of symbolic control, there is no direct, mimetic relationship between the actual movement that is performed by the player and the corresponding movement in-game, executed by the avatar. Actions like running and jumping are not controlled through real life movements; players simply press symbolic buttons that they know to correspond with but are not strongly related to the actions in-game (Calleja, 2011; Skalski et al., 2011).

On the other end of the spectrum, there is symbiotic control, in which the player’s physical movements in real life are detected and mapped onto the avatar and have a close relationship with the virtual response of the avatar in the game world (Calleja, 2011; Skalski et al., 2011; Vanden Abeele, 2011). The best example of symbiotic control is the relatively new and popular *Microsoft Kinect* interface, which can be used with *Microsoft Xbox 360 *consoles and *Microsoft Windows *PCs. This type of control is substantially different from the pressing of symbolic buttons, since players have to physically move themselves in order to cause the appropriate action in the game (Calleja, 2011). *Kinect* utilizes a camera that is attached to the television or PC and maps player movement directly onto the avatar. If people are playing a fighting game, for instance, they will have to use their entire body to punch and kick their foes (Calleja, 2011).

Finally, a milder version of symbiotic control is mimetic control, which constitutes a partial mapping of the player’s actions onto the avatar. Well-known and popular examples of mimetic controllers include the *Nintendo Wii Remote* and the *Sony* *PlayStation Move *motion**controller. Only the movement of these motion-sensing controllers is registered, so players have to swing the controller in the wanted direction and with the wanted intensity, triggering a similar response in-game (e.g., swinging the controller as a baseball bat or pointing it as a gun). Another form of mimetic control can be found in controllers that replicate part of a machine, tool, vehicle, or instrument (e.g., steering wheels, light guns, musical instruments; Calleja, 2011; Skalski et al., 2011).

Apart from these different manifestations of game control, a player’s sense of kinesthetic involvement is highly dependent on the perceived difficulty of the controls. When people start playing a game, they go through an entire process that ranges from the learning of the game controls to the automation of control and movement (Calleja, 2011). When a player is not yet sure which key corresponds to a certain action, he needs to direct more of his attention to the key presses, learning by trial and error and sometimes even needing to consult a guide. After a while, however, the controls will be learned and practiced to such a degree that the player will be able to press the keys automatically, resulting in the on-screen movement feeling unmediated and the player being able to direct his attentional resources to other aspects of the game (Calleja, 2011). This is especially relevant for the practice of IGA: as players increasingly learn controls, they can devote more of their attention to the exploration of their surroundings, including the advertising-related elements they feature.

In summary, kinesthetic involvement is a crucial part of the gaming experience, as most other aspects of involvement in games are dependent on developing at least a basic fluency of movement in the environment. Therefore, in order to be able to elaborate on the impact of player involvement on the effectiveness of advertising featured in a digital game, we decided to conduct an experimental study in which we were primarily interested in the effect of kinesthetic involvement. In this experiment, we varied players’ control over the game by manipulating the *type of game controller* with which the game was played as a *within-subjects factor*, resulting in two experimental conditions. We chose to work with a racing game in the experiment, since racing games are very performative games in which the player has to constantly execute kinesthetic actions, manipulating the controller while following the visual cues shown on the screen (Apperley, 2006). In one condition, respondents played the racing game with a symbolic game controller (i.e., a traditional gamepad controller), while in the other condition, participants played the racing game with a mimetic controller (i.e., a motion-based racing wheel controller). The choice for a *traditional, symbolic controller versus a motion-based mimetic one* was made because prior research studying player experience had already shown that these types of controllers lead to significant differences in player involvement, especially affecting a person’s sense of kinesthetic involvement (e.g., Johnson et al., 2002; Limperos et al., 2009; Skalski et al., 2011; Vanden Abeele, 2011). Moreover, they can also influence the way in which people process IGA, as shown by a study of Dardis et al. (2012). In what follows, we will give an overview of relevant literature concerning the effects of game controller type on kinesthetic involvement on the one hand and the processing of IGA in terms of brand awareness on the other hand, and formulate hypotheses accordingly. Moreover, we will discuss the mechanisms that possibly underlie the effects of game controller on IGA memory and make a case for the mediating role of kinesthetic involvement.

#### The impact of game controller on kinesthetic involvement

First of all, studies looking at the impact of game controller on the player experience show that playing games with a motion-based, mimetic game controller augments players’ perceived controller naturalness (Skalski et al., 2011; Vanden Abeele, 2011). Since these game controllers exploit a direct relation between the physical actions of the gamer and the in-game actions of the avatar, they are perceived as being more predictable, logical, intuitive, and natural (Skalski et al., 2011; Vanden Abeele, 2011). However, intuitiveness and control are two different things. Although it is often believed that motion-based play is easier than playing with a traditional, symbolic game controller, research proves that the opposite is often the case (Johnson et al., 2002; Vanden Abeele, 2011). Although physical controllers allow for more intuitive and natural game controls, they also still suffer from a lack of precision and responsiveness, making it harder for players to control their actions and movements in the game environment (Johnson et al., 2002; Vanden Abeele, 2011). Studies from Vanden Abeele (2011) and McMahan et al. (2010) also find that motion-based controllers not only decrease perceived control but also actual control, resulting in a lower game performance (e.g., lower game scores, slower game completion times).

Based on Calleja’s (2011) description of the kinesthetic involvement dimension and the results of the studies mentioned above, we deem kinesthetic involvement to be influenced by and thus to consist of two sub dimensions. The first sub dimension concerns the player’s *control* over his actions and movements in the game world and is highly dependent on the perceived difficulty of the game controls, while the second sub dimension is related to the perceived *naturalness* of the game controller. We expect that our manipulation of game controller type (traditional symbolic controller versus motion-based mimetic controller) will affect both sub dimensions; we predict that the mimetic controller will be perceived as more intuitive and natural compared to the symbolic controller, but that it will also be less precise and responsive, making it harder for players to control the game. As such, we propose the following hypotheses:

H1: *People will experience higher levels of control over their actions and movements in-game when playing the game with the symbolic controller compared to playing with the mimetic controller.*

H2: *People will experience lower levels of controller naturalness when playing with the symbolic controller compared to playing with the mimetic controller.*

Moreover, we will also look at the impact of game controller type on the players’ actual game performance, expecting the following:

H3: *People will have a higher game performance when playing with the symbolic controller compared to playing with the mimetic controller.*

Finally, we will examine the influence of game controller type on kinesthetic involvement in general. Playing with the mimetic controller is suspected to lead to higher levels of perceived controller naturalness, while the symbolic controller is hypothesized to be easier to command, leading to increased responsiveness and control over the game world. We expect that when considering kinesthetic involvement in its entirety, control will carry more weight than naturalness, since it puts a greater strain on the player’s attention. Consequently, we formulate the following hypothesis:

H4: *People who play the game with the symbolic controller will experience higher levels of kinesthetic involvement than those who play the game with the mimetic controller.*

#### The impact of game controller on brand awareness

If the manipulation of game controller is indeed able to cause significant variances in kinesthetic involvement, we are interested in analyzing whether it also affects the way people process the advertisements integrated into the game environment.

Concerning the influence of game controller on people’s awareness of the brands integrated into the game, we start from the assumptions of the LC4MP (Lang, 2009). The LC4MP states that a person’s ability to process information is limited, with people only having access to a limited pool of cognitive resources at a particular time (Lang, 2009). In the context of our study, this model has important implications for the processing of IGA. Digital games are highly interactive and involving media that bombard the player with a continuous stream of sensory (i.e., audiovisual, tactile) information. Getting an advertisement noticed and remembered in such an involving game environment is not self-evident (Grigorovici and Constantin, 2004; Lee and Faber, 2007). People allocate their attentional resources to those aspects of a task or activity that are most relevant to them at a particular time (i.e., the primary task). In a digital game context, the primary task consists of actually playing of the game; the player tries to process, remember and act on the information that is most essential for his progression in the game. Since people will focus their attention primarily on the playing of the game, this leaves fewer mental resources available for secondary tasks such as the processing of advertisements that are embedded into the game (Grigorovici and Constantin, 2004; Lee and Faber, 2007; Lang, 2009). The LC4MP (Lang, 2009) will thus form the basis for our hypotheses to be built upon.

The influence of game controller on the processing of IGA has been studied in one previous study before. This study from Dardis et al. (2012) showed that variations in game controller can indeed reflect on the memory of the ads featured in a game; playing a racing game with a symbolic controller (i.e., *Xbox 360* gamepad controller) resulted in increased ad recall rates compared to playing with a mimetic controller (i.e., *Xbox 360* racing wheel with gas and brake pedals). They, too, explain these findings by referring to the LC4MP (Lang, 2009), suggesting that players are more familiar with the traditional symbolic controllers compared to the newer mimetic controllers, which therefore require less attentional resources. However, they do not explicitly measure players’ familiarity or expertise with the game controllers, leaving the mechanisms that underlie the effect of game controller open for discussion.

***Kinesthetic involvement as mediator.*** The aim of our study, then, is to further investigate and verify the effect of game controller on the processing of IGA (i.e., brand awareness: brand recall and brand recognition), and determine whether or not they can be explained by variations in kinesthetic involvement.

Previous research already established that player involvement is a relevant factor to consider when studying IGA, showing that fluctuations in a person’s general absorption or involvement in a digital game can alter the processing of IGA (e.g., Grigorovici and Constantin, 2004; Lee and Faber, 2007). Grigorovici and Constantin (2004), for instance, investigated the influence of involvement on the awareness of IGA in a virtual environment. Their results show that the more involving a virtual environment is, the lower people’s brand recall and recognition. Lee and Faber (2007) looked at effects of involvement while playing an online racing game on brand memory, and also found that it limited players’ awareness of the brands integrated into the game. Both studies clarify these effects on brand awareness by quoting the LC4MP (Lang, 2009), arguing that highly involving environments put an increased strain on people’s cognitive resources, resulting in people devoting their attention more to playing the game and less to the processing of IGA (Grigorovici and Constantin, 2004; Lee and Faber, 2007; Lang, 2009).

The findings of these studies – looking at both the impact of game controller (Dardis et al., 2012) and player involvement (Grigorovici and Constantin, 2004; Lee and Faber, 2007) on IGA processing – underline the importance of considering the multidimensional nature of player involvement. Following the reasoning of Dardis et al. (2012), we suspect that in the specific case of kinesthetic involvement, results might differ from studies looking at general involvement, although we expect them to still be in line with the reasoning of the LC4MP (Lang, 2009). As we mentioned before, kinesthetic involvement can range from the learning of new controls, to the automation of control and movement in a game. As a player becomes more practiced and familiar with the game controls, he will be able to press the buttons automatically, without paying conscious attention to them. His attentional resources can therefore be directed to other aspects of the game, such as the advertisements integrated into the game environment (Calleja, 2011). Since we hypothesized that playing with a symbolic controller will be easier, increasing the player’s control over the game and the sensation of movement it produces, we propose the following hypothesis:

H5: *People will experience higher levels of brand awareness (recall, recognition) when playing with the symbolic controller compared to playing with the mimetic controller.*

Finally, if our results show that the manipulation of game controller indeed has a significant influence on participants’ awareness of the brands encountered in-game, we expect that this effect will be mediated by their sense of kinesthetic involvement.

H6: *The sub dimensions of kinesthetic involvement will mediate the relationship between type of game controller (symbolic controller versus mimetic controller) and brand awareness (recall, recognition).*

### BRAND PROMINENCE

Further, we want to analyze the influence of an additional factor that might affect the impact of IGA: the prominence of the brand placement. Brand prominence depends on placement characteristics such as ad size, color, attractiveness, and spatial position. These characteristics are of considerable importance in an advertising context. Advertising studies investigating effects in traditional media (e.g., television, print) have demonstrated that the placement of a brand in a prominent way generally has a positive effect on brand memory, since a prominent ad attracts more attention and is more deeply processed resulting in increased awareness (Law and Braun, 2000; Van Reijmersdal, 2009). In an IGA context, several studies have already looked at the effect of brand prominence on brand awareness, although they mostly focused on only one placement characteristic (e.g., Grigorovici and Constantin, 2004; Acar, 2007; Lee and Faber, 2007; Bardzell et al., 2008; Jeong and Biocca, 2012) or on all characteristics at the same time (e.g., Schneider and Cornwell, 2005). Their findings reveal that prominent placements (e.g., large versus small placements, central versus peripheral placements) indeed lead to higher levels of recall and recognition compared to more subtle placements.

In the current study, we aim to elaborate on the effect of brand prominence by examining how different combinations of ad size and spatial position affect people’s response to the brands featured in IGA in terms of recall and recognition. Therefore, we manipulated the size (small versus large) and spatial position (peripheral versus central) of the in-game ads integrated into the experimental game, resulting in four different placement types (large-central, small-central, large-peripheral, small-peripheral). We expect that highly prominent brand placements will lead to higher brand awareness rates compared to more subtle placements. In our experiment, the large-central brands can be considered to be the most prominent, while small-peripheral placements are the most subtle.

H7: *Large-central brand placements will obtain higher levels of brand awareness (recall, recognition) compared to small-peripheral placements.*

However, our results will have to point out which placement characteristics (ad size or spatial position) prove to be the most important in light of IGA effectiveness, leading us to formulate the following research question:

RQ1: *Which combinations of placement characteristics (size x spatial position) are the most effective in terms of brand awareness (recall, recognition)?*

Finally, we also want to check whether the effects of our manipulations of game controller and brand prominence interact with each other:

RQ2: *Do the effects of game controller (symbolic controller versus mimetic controller) and brand prominence (size x spatial position) on brand awareness (recall, recognition) interact with each other?*

## METHOD

### EXPERIMENTAL DESIGN

To be able to test the impact of kinesthetic involvement on the effectiveness of IGA, we conducted an *experiment* with a *within-subjects design*. During this experiment, participants played a *Sony PlayStation 3* kart racing game containing several in-game advertisements. In order to vary kinesthetic involvement, we manipulated the players’ control over the game between two conditions by letting them play the game twice, with two *different types of* *game controllers*. In one condition, participants played the game with the *traditional PlayStation 3 controller* (i.e., a gamepad or symbolic game controller), while in the other condition, they played the game with the *PlayStation Move racing wheel* (i.e. a mimetic game controller that combines the motion-sensing abilities of the *PlayStation Move* controller with a steering wheel). The order in which participants played with the two different controllers was counterbalanced to avoid order effects.

Additionally, in order to elaborate on the influence of *brand prominence*, we manipulated the *size* (small versus large) and *spatial position* (peripheral versus central) of the in-game ads integrated into the game, combining both characteristics into four different placement types (large-central, small-central, large-peripheral, small-peripheral).

### PARTICIPANTS

Thirty one people (24 male, seven female), 18 to 30 years of age (*M *= 22.6, *SD *= 2.9) participated in the experiment. Although people were only required to have basic experience with games in order to be able to participate, the majority of our sample can be considered experienced gamers. All participants had been playing digital games for 6 years or more (6 to 8 years: 12.9%, 9 years or more: 87.1%), and most of them played games on a weekly or daily basis (a few times a year: 12.9%, monthly: 9.7%, weekly: 48.4%, daily: 29.0%).

### EXPERIMENTAL GAME AND IN-GAME ADVERTISEMENTS

The game *LittleBigPlanet Karting*^2^ was used in the experiment. *LittleBigPlanet Karting *is a *Sony* *PlayStation 3*-exclusive kart racing game in which players race against computer opponents or other players in a go-kart on a variety of tracks. Throughout the race, players can pick up both offensive and defensive weapons to either attack or protect themselves from their opponents. Moreover, the game focuses heavily on user-generated content, enabling us to create our own levels using the official editor of the game. We made two levels for use in the experiment: one level for each condition. Since we wanted to analyze the effect of controller type on the effectiveness of IGA, these two levels looked exactly the same except for the integration of the advertising in the game environment. The race track that we created was set in a village environment and players had to complete five racing laps, competing against seven computer opponents. Players did not have a time limit, but since it was a racing game their goal was to finish the game level in the best time and placing possible.

We chose to incorporate billboard advertisements inside our levels. Billboard ads are one of the most common forms of advertising in racing games and the experimental setup thus resembled the real-life practice of IGA (Nelson, 2005; Skalski et al., 2010). As we wanted to investigate the impact of ads with varying sizes (large versus small) and spatial positions (central versus peripheral), we combined both ad characteristics into four different placements: large-central, small-central, large-peripheral, and small-peripheral. The peripheral billboards were placed on the side of the road, while the central billboards were attached to bridges the player had to drive underneath, featuring the placements in the center of the screen. The logos of the brands featured on the large billboards were exactly the same size, as were the logos of the brands on the small billboards. Each racing lap featured these four placements, meaning that players encountered each placement five times.

**FIGURE 1:**
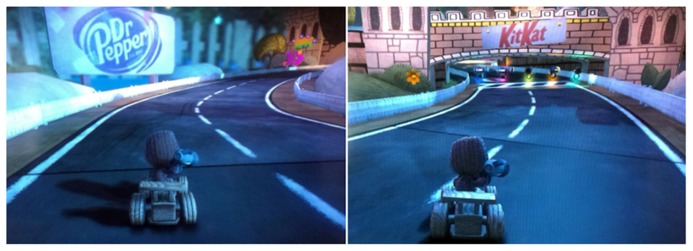
**Pictures of advertisements (i.e., billboards) in the experimental game: large-peripheral placement (left), small-central placement (right)**.

The brands that were featured on the in-game billboards were popular and well-known soda and candy brands. We chose to work with existing and well-known brands in order to create a realistic and plausible IGA scenario. As already mentioned, we created two game levels for use in the experiment. These two levels looked exactly the same, apart from the integration of IGA. Each level incorporated four different brands that were found to be similar in familiarity and attitude based on the results of a *pretest* with 43 people (32 male, 11 female, *M *= 22.2, *SD *= 4.0), in order to be able to distinguish the impact of the manipulation of game controller on IGA effectiveness (see **Table 1**). Again, the order in which the participants played the levels with the different types of game controllers was counterbalanced to avoid order effects. By making use of the *PlayStation Eye* camera, we transferred the real logos of these brands onto the billboards that were placed into the game (see **Figure 1**).

**Table 1:** Overview of the brands featured in the two experimental levels.

Brand prominence	Level 1				Level 2			
		Familiarity	Attitude			Familiarity	Attitude	
		*%*	*M*	*SD*		*%*	*M*	*SD*
**Large-central**	*Twix*	100	5.233	1.269	*M&M’s*	100	5.140	1.226
**Small-central**	*Snickers*	100	4.837	1.526	*Kit Kat*	100	4.814	1.139
**Large-peripheral**	*Canada Dry*	95.3	5.000	1.225	*Dr. Pepper*	95.3	4.421	1.407
**Small-peripheral**	*Schweppes*	97.7	4.452	1.131	*Orangina*	97.7	4.595	1.127

**Note: *Brand attitude was measured on a scale from 0 (very negative) to 7 (very positive).*

*Brand familiarity indicates the percentage of the respondents that were familiar with the brand.*

*The differences in familiarity [*F*(7,294) = 1.103, *NS*] and attitude [*F*(7,259) = 1.984, *NS*] between the brands were non-significant.*

### PROCEDURE

The experiment took place in a lab room at the University of Antwerp (Belgium). In this game lab we had a *Sony PlayStation 3* console at our disposal, connected to a large television screen. During the experiment, participants first played the official tutorial of the game with one controller [either the symbolic controller (i.e., traditional *PlayStation 3* controller) or the mimetic controller (i.e., *PlayStation Move* racing wheel)], explaining how to play the game (e.g., steer, pick up weapons). Afterward, they played the experimental game level containing IGA. When they finished playing, we wrote down their game score and game completion time and they had to fill out the first part of a self-report questionnaire, asking them about their player involvement while playing the game with the specific game controller. When they completed this questionnaire, the second part of the experiment started, and they had to repeat the whole process with the other game controller: play tutorial – play experimental level – fill out player involvement questionnaire. The order in which participants played these experimental game levels with the two different controllers was counterbalanced to avoid order effects. Finally, after playing the game twice and filling out the player involvement questionnaires, respondents were asked to fill out a questionnaire exploring the effectiveness of in-game ads, and their socio-demographic and gamer characteristics. Participation in the experiment took approximately 45 min.

### MEASURES

#### Game performance

We measured participants’ *game scores* with each game controller (i.e., the place they finished in the race), as well as the *time* it took them to complete the racing level.

#### Self-report measures

***Kinesthetic involvement.** Kinesthetic involvement* was measured by 14 items (*Cronbach’s α* = 0.933), covering the player’s involvement generated by both the controllability and naturalness of the game controllers. Twelve items gauged the players’ perceived control over their actions and movements of the avatar in-game, and to which extent they found the controls easy to use (e.g., “I felt that my avatar was responsive to actions that I initiated,” “I felt proficient in moving around in the game environment,” “The game controls were easy to pick up”), while the two other items measured the perceived naturalness and intuitiveness of the game controllers (e.g., “The actions necessary for controlling the game were very close to that in the real world”). Agreement with these items was measured on a five-point intensity scale ranging from “not at all” (0) to “extremely” (4). The utilized scales were based on Witmer and Singer’s (1998) presence questionnaire, Jennett et al.’s (2008) immersion scale, and Vanden Abeele’s (2011) perceived control and perceived controller naturalness scales.

A principal component analysis (PCA) was conducted on these 14 items with oblique rotation (direct oblimin). The Kaiser–Meyer–Olkin measure verified the sampling adequacy for the analysis (KMO = 0.932). Bartlett’s test of sphericity χ ^2^ (91) = 938.563, *p* < 0.001, indicating that correlations between items were sufficiently large for PCA. Results demonstrate that two components had eigenvalues that were greater than 1 and in combination explained 78.343% of the variance. These two components were in line with our expectations and revealed the *control* versus *controller naturalness* sub dimensions of kinesthetic involvement. **Table 2** shows the factor loadings after rotation. Next, we averaged the scores of the items loading on these two factors, leading to two new variables [*control (Cronbach’s α* = 0.97) and *controller naturalness* (*Cronbach’s α* = 0.89)].

**Table 2:** Summary of the principal component analysis results of kinesthetic involvement.

Kinesthetic involvement	Rotated factor loadings		
Item	Control		Controller naturalness
I felt that my avatar was responsive to actions that I initiated	0.842		
The game reacted exactly as I wanted to my actions	0.894		
I did not feel as if I was moving through the game according to my own will (-)	0.612		
I felt proficient in moving around in the game environment	0.948		
I felt in control when playing the game with the game controller	0.927		
I could control the game flawlessly	0.919		
I thought the game was easy to control	0.916		
The game controls were easy to learn	0.784		
I became unaware that I was even using any game controls	0.833		
I could concentrate on the assigned tasks or required activities rather than on the game controls used to perform those tasks or activities	0.919		
Controlling the game was difficult (-)	0.877		
The game controls were easy to pick up	0.841		
The actions necessary for controlling the game were very close to that in the real world			0.935
Controlling the game world felt very much like controlling in the real world			0.933
**Eigenvalues**	9.217		2.717

**% Variance **		78.343
**Component correlation**		-0.281

**Note: *Factor loadings of less than 0.200 have been omitted.*

***In-game advertising effectiveness.** Brand awareness* was measured on three levels. First of all, participants were asked to spontaneously recall which brands they remembered encountering in the digital game (i.e., free recall). Subsequently, participants were presented with a list of brand names (i.e., brand name recognition), and a list of brand logos (i.e., brand logo recognition). In each case, participants had to indicate which brand names and brand logos they remembered seeing in-game. For each recognition measure, the four correct options were included, as well as eight filler items and an “I don’t know” option. The data that originated from these measures were combined into brand awareness variables that indicate how many brands (names, logos) each participant correctly recalled or recognized (*brand recall*, *brand name recognition*, *brand logo recognition*).

We measured IGA effectiveness in terms of *brand evaluation* as well, i.e., brand attitude and purchase intention. *Brand attitudes* of the integrated brands were measured by the mean of three seven-point scales anchored by the adjectives “good (0) – bad (6)”, “like very much (0) – dislike very much (6),” and “pleasant (0) – unpleasant (6)” (*Cronbach’s α* values range from.95 to.98). *Purchase intentions* of the brands were measured by using a four-point scale going from “not at all likely to buy” (1) to “very likely to buy” (4). However, we did not expect to find an effect of our manipulations on brand attitude or purchase intention, since participants only played the game for a very short while. Moreover, the game featured well-known, popular brands in order to create a realistic encounter with IGA. We thought it unlikely that the limited exposure to IGA (due to the short playing duration) would lead to significant changes in the evaluations of these established brands. Our findings show that this is indeed the case: the manipulations did not lead to significant changes in attitudes or the intention to buy the products. Therefore, we do not include the results concerning brand evaluation in the current paper and focus mainly on IGA effectiveness in terms of brand awareness.

***Background information.*** Finally, participants were asked about their *socio-demographic characteristics* (e.g., gender, age) and their *gamer profile* (e.g., game experience, frequency, and familiarity with the two game controllers that were used (i.e., traditional *PlayStation 3* gamepad controller and *PlayStation Move* racing wheel controller). These variables were tested for their potential moderating effects, but were not found to be significant moderators.

## RESULTS

### THE IMPACT OF GAME CONTROLLER

As mentioned before, we conducted a within-subjects experiment in which we manipulated the type of game controller that the players used to command the game, resulting in two conditions [symbolic controller (i.e., traditional *PlayStation 3* controller) versus mimetic controller (i.e., *PlayStation Move* racing wheel)]. In order to test our hypotheses in this within-subjects context, we performed one-way repeated measures analyses of variance (ANOVAs) to examine the impact of game controller on (1) kinesthetic involvement and game performance and (2) the processing of IGA in terms of brand awareness (i.e., brand recall, brand name recognition, brand logo recognition).

First of all, we looked at the effect of the manipulation of game controller on the players’ sense of kinesthetic involvement. We have defined *kinesthetic involvement* as consisting of two sub dimensions: *control* and *controller naturalness*. The existence of these two sub dimensions was confirmed by the principal component analysis we performed (see **Table 2**). One-way repeated measures ANOVAs show that there are significant differences in all of these sub dimensions of kinesthetic involvement between experimental conditions (see **Table 3**). Playing the game with the symbolic controller led to more control over actions and movements in-game [*F*(1,30) = 90.816, *p* < 0.001], but playing the game with the mimetic controller was perceived as more natural [*F*(1,30) = 26.774, *p* < 0.001]. These results are therefore in line with *hypotheses 1* and *2*.

**Table 3:** The impact of game controller on kinesthetic involvement.

Game controller	Kinesthetic involvement								
	Control			Controller naturalness			Total		
	*Mean*		*SD*	*Mean*		*SD*	*Mean*		*SD*
**Symbolic**	2.916		0.610	0.645		0.848	2.385		0.545
**Mimetic**	1.495		0.695	1.694		0.980	1.578		0.673

***F*(1,30)**		90.816*			26.774*			33.757*

Note: *p < 0.001.

*The sub dimensions of kinesthetic involvement were measured by using five-point intensity scales ranging from 0 to 4.*

Moreover, when looking at the impact of game controller on game performance (i.e., *game scores* and *game completion time*) we see that the traditional game controller does not only lead to higher perceived control, but also to higher actual control. Results of one-way repeated measures ANOVAs demonstrate that playing with the mimetic controller has a detrimental effect on players’ game score or the place in which they finished [*F*(1,30) = 34.634, *p* < 0.001; *M*_symbolic controller_ = 2.032, *SD* = 2.213, *M*_mimetic controller_ = 5.258, *SD* = 2.932] and on the time (measured in seconds) in which players finished the race [*F*(1,30) = 92.540, *p* < 0.001; *M*_symbolic controller_ = 330.516, *SD* = 20.805, *M*_mimetic controller_ = 366.065, *SD* = 18.417]. This result is thus in line with the expectations formulated in *hypothesis 3*.

Finally, when taking into account *kinesthetic involvement in its entirety*, we see that control outweighs naturalness (see **Table 3**): playing with the symbolic controller results in higher levels of kinesthetic involvement compared to playing with the mimetic controller [*F*(1,30) = 33.757, *p* < 0.001], providing support for *hypothesis 4*.

Next, we looked at the impact of game controller manipulation on the effectiveness of IGA in terms of *brand awareness* (i.e., *brand recall, brand name recognition, *and* brand logo recognition*). The findings of one-way repeated measures ANOVAs point out that there are significant variations in the recall and recognition rates of the brands between conditions: playing the game with the symbolic controller results in significantly higher levels of brand recall [*F*(1,30) = 13.304, *p* < 0.001], brand name recognition [*F*(1,30) = 40.208, *p* < 0.001] and brand logo recognition [*F*(1,30) = 38.958, *p* < 0.001] compared to playing with the mimetic controller (see **Table 4**). These results support *hypothesis 5*.

**Table 4:** The impact of game controller on brand awareness.

Game controller	Brand awareness								
	Brand recall			Brand recogn.			Logo recogn.		
	*Mean*		*SD*	*Mean*		*SD*	*Mean*		*SD*
**Symbolic**	1.000		0.817	1.677		0.909	1.968		0.912
**Mimetic**	0.355		0.615	0.548		0.723	0.807		0.750

***F*(1,30)**		13.304*			40.208*			38.958*

Note: *p < 0.001.

*The brand awareness variables indicate how many brands each participant correctly recalled or recognized (0–4) in each experimental condition.*

### THE IMPACT OF KINESTHETIC INVOLVEMENT

The results thus show that the manipulation of game controller has a significant influence on (1) players’ sense of involvement with the kinesthetic properties of the game and (2) the effectiveness of IGA in terms of brand awareness.

In order to check whether kinesthetic involvement mediates the impact of game controller on brand awareness, the direct effect of kinesthetic involvement on brand awareness was subsequently examined. Linear mixed model analyses on the brand awareness variables with the sub dimensions of kinesthetic involvement as repeated measures factors reveal that *brand recall* is not significantly affected by either of the sub dimensions. Both of the recognition measures, however, are significantly influenced by control (effect on *brand name recognition*: *F*(33,28.000) = 2.507, *p* = 0.008; *brand logo recognition*: *F*(33,28.000) = 3.002, *p *= 0.002).

Next, we performed linear mixed model analyses on the brand awareness variables with game controller type as factor and the control sub dimension of kinesthetic involvement as a repeated measures covariate (i.e., mediation analyses). Regarding *brand name recognition*, we see that the effect of game controller is weakened [*F*(1,59.000) = 4.096, *p* = 0.048] when control is included, although its mediating effect is not able to reach significance [*F*(1,59.000) = 3.145, *NS*]. When looking at the effect on *brand logo recognition*, the impact of game controller is diminished to the point of non-significance [*F*(1,59.000) = 2.966, *NS*], with control [*F*(1,59.000) = 5.174, *p* = 0.027] serving as a significant mediator. These results partially support *hypothesis 6*, showing that players’ perceived control over their actions and movements in-game underlie the impact of game controller on the awareness of IGA.

### THE IMPACT OF BRAND PROMINENCE

In order to analyze the influence of brand prominence, we included the placement characteristics of *ad size* (large versus small) and *spatial position* (central versus peripheral) as an additional within-subjects factor in our experiment, resulting in four different placements (large-central, small-central, large-peripheral, small-peripheral). Since we created two levels for use in the experiment, each with placements for four different brands, we first of all checked whether the matched brands (e.g., large-central brand in level 1 versus large-central brand in level 2) differed significantly from each other in terms of brand awareness. One-way repeated measures ANOVAs on the awareness variables of the matched brands show that the differences were non-significant for all placement types, allowing us to combine the awareness scores of the brands into average brand awareness variables per placement.

To test the effect of brand prominence on brand awareness, we performed one-way repeated measures ANOVAs on the brand awareness rates of these four types of placements. Concerning *brand recall*, results reveal that there are significant differences between the different placements [*F*(3,90) = 3.425, *p* = 0.021]. Large-central placements (*M* = 0.548, *SD* = 0.568) obtain the highest recall rates, followed by small-central placements (*M* = 0.387, *SD* = 0.615), large-peripheral placements (*M* = 0.258, *SD* = 0.445), and lastly, small-peripheral placements (*M* = 0.161, *SD* = 0.374). Bonferroni *post hoc* tests demonstrate that the significant differences are situated between the large-central and small-peripheral placements (*p* = 0.003).

Regarding *brand name recognition*, results are similar. The different placement types differ significantly in their effect on brand name recognition [*F*(3,90) = 7.035, *p* < 0.001], with large-central placements (*M* = 0.871, *SD* = 0.562) having the greatest influence, followed by small-central (*M* = 0.645, *SD* = 0.709), large-peripheral (*M* = 0.452, *SD* = 0.568), and small-peripheral placements (*M* = 0.258, *SD* = 0.445). Bonferroni *post hoc* tests reveal that the large-central placements are again significantly different from the small-peripheral placements (*p* < 0.001).

Finally, when looking at *brand logo recognition*, results show that the different placement types also vary significantly in their effect [*F*(3,90) = 7.520, *p* < 0.001], with large-central placements (*M* = 1.032, *SD* = 0.547) having the greatest impact, followed by the small-central (*M* = 0.807, *SD* = 0.703), large-peripheral (*M* = 0.548, *SD* = 0.624) and the small-peripheral placements (*M* = 0.387, *SD* = 0.495). Bonferonni *post hoc* tests demonstrate that the large-central placements vary significantly from the large-peripheral (*p* = 0.030) and small-peripheral placements (*p* < 0.001).

Based on these results, we can accept *hypothesis 7*: the most prominent (i.e., large-central) placements obtain significantly higher rates of awareness compared to the most subtle (i.e., small-peripheral) placements. Moreover, we can answer *research question 1*: when looking at the effectiveness of different types of IGA placements in terms of ad size and spatial position, results indicate that especially spatial position is of importance, with the central placements obtaining the highest recall and recognition scores. The effect of ad size is much smaller; large placements are not able to lead to significant differences in brand awareness compared to their smaller counterparts.

**FIGURE 2:**
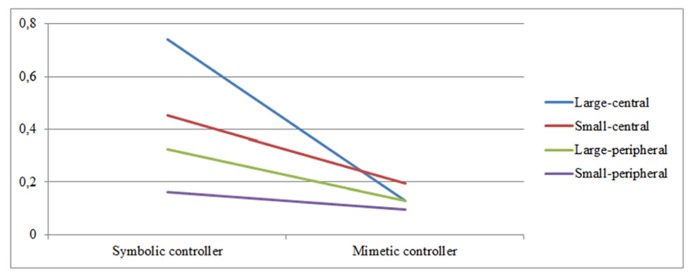
**Interaction effects of game controller and brand prominence on brand name recognition**.

Lastly, in order to be able to answer *research question* *2*, we checked for interaction effects of our manipulations on the awareness of IGA by conducting two-way repeated measures ANOVAs and including game controller type and brand prominence as within-subject factors. Our results show a significant interaction effect of game controller and brand prominence on *brand name recognition* [*F*(3,90) = 5.016, *p* = 0.003]. When looking at the results in greater detail, we see that game controller significantly affects the brand name recognition of the large-central [*F*(1,30) = 37.345, *p* < 0.001] and small-central placements [*F*(1,30) = 6.234, *p* = 0.018] while the recognition rates of the peripheral placements are not affected. Moreover, we observe significant changes in brand name recognition between the different placement types when people are playing with the symbolic controller [*F*(1,30) = 9.158, *p* < 0.001], but not with the mimetic controller (see **Figure 2**).

**FIGURE 3:**
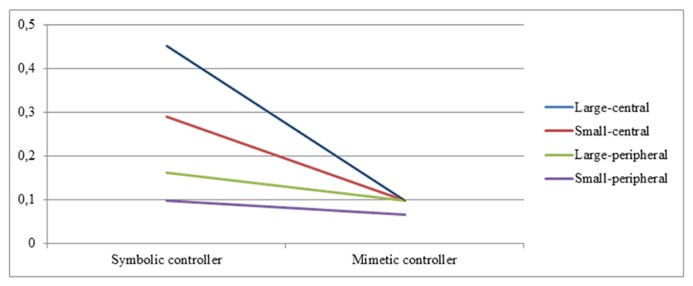
**Interaction effects of game controller and brand prominence on brand recall (non-significant)**.

The results concerning *brand recall* show a similar trend: game controller significantly affects the recall rates of the large-central [*F*(1,30) = 10.552, *p* = 0.003) and small-central placements [*F*(1,30) = 5.094, *p* = 0.031], while the recall rates of the peripheral placements are not affected. We also observe significant changes in recall between the different placement types when playing with the symbolic controller [*F*(1,30) = 4.288, *p* = 0.007], but not with the mimetic controller (see **Figure 3**). However, this interaction effect of game controller and brand prominence is not able to reach significance [*F*(3,90) = 2.553, *NS*].

**FIGURE 4:**
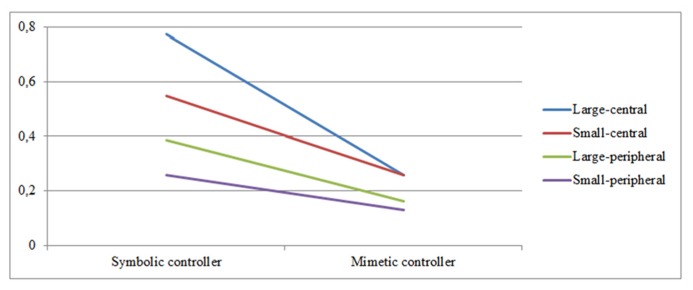
**Interaction effects of game controller and brand prominence on brand logo recognition (non-significant)**.

Finally, results show that the interaction effect of game controller and brand prominence on *brand logo recognition *is also not significant**[*F*(3,90) = 1.842, *NS*]. Here, the manipulation of game controller significantly affects all placements [large-central [*F*(1,30) = 18.028, *p* < 0.001], small-central [*F*(1,30) = 6.328, *p* = 0.017], and large-peripheral placements [*F*(1,30) = 4.153, *p* = 0.050]] except for the small-peripheral ones. The brand logo recognition rates of the different placement types also significantly differ when playing with the symbolic controller [*F*(1,30) = 6.857, *p* < 0.001], although they still do not when playing with the mimetic controller (see **Figure 4**).

## DISCUSSION

The aim of the study was to contribute to research on the effectiveness of IGA by analyzing the impact of two contextual factors on the processing of IGA, namely a person’s sense of *involvement* in a game, and the *prominence* of the advertisements that are integrated into the game.

Prior research had already established that *player involvement* is a relevant factor to consider in an IGA context, showing that different levels of a player’s general involvement with a game affect the way they process IGA in terms of brand awareness (e.g., Grigorovici and Constantin, 2004; Lee and Faber, 2007). However, involvement is a multidimensional construct and in the specific context of digital games, it is understood as a combination of six dimensions that are able to capture the player’s attention (Calleja, 2011). The study scrutinizes the effects of one of these dimensions, namely *kinesthethic involvement* or the player’s involvement related to the modes of control and movement in a game (Calleja, 2011).

In order to test the specific effect of kinesthetic involvement on the processing of IGA, we conducted a *within-subjects experiment* in which we manipulated the type of *game controller* that was used to play the game between two conditions [symbolic controller (i.e., traditional *PlayStation 3* gamepad controller) versus mimetic controller (i.e., motion-based *PlayStation Move* racing wheel)]. Results show that this manipulation of game controller had a significant impact on the players’ sense of involvement with the kinesthetic properties of the game. The controls of the traditional symbolic controller were easier to learn and handle, allowing the players more precise control over their movements and actions in the game world, while the controls of the motion-based mimetic controller were perceived to be more natural and intuitive. These results replicate the findings of previous studies looking at the effects of game controller type on player experience and performance (Johnson et al., 2002; McMahan et al., 2010; Skalski et al., 2011, Vanden Abeele, 2011). When looking at kinesthetic involvement in its entirety, we see that control outweighs naturalness: playing the game with the symbolic controller led to higher levels of kinesthetic involvement compared to playing the game with the mimetic controller.

Moreover, the variation in game controller significantly influenced the processing of the advertisements embedded in the game: when participants played the game with the symbolic controller, they were able to recall and recognize significantly more brands. This finding is in line with the results from Dardis et al. (2012), who also found that playing with a symbolic (versus mimetic) game controller led to increased ad recall scores.

We subsequently looked at the impact of the sub dimensions of kinesthetic involvement on brand awareness. Results show that brand recall was not significantly influenced by kinesthetic involvement. However, both of the brand recognition measures were significantly affected by a person’s perceived control over his actions and movements in the game. Mediation analyses further showed that the impact of game controller on brand logo recognition was fully mediated by the player’s perceived control. This finding suggests that when a game is easier to control, the process going from the learning of the controls to the automation of movement in-game will happen more quickly, freeing attentional resources, and leading to people being able to pay more attention to secondary elements of the game such as IGA. These results are therefore in line with the LC4MP (Lang, 2009).

Apart from the influence of game controller type and kinesthetic involvement, we additionally checked the impact of *brand prominence* in an IGA context. Brand prominence is a factor that depends on several placement characteristics such as ad size, color, attractiveness, and spatial position. Several studies already looked at the effect of these placement characteristics, showing they can have a major impact on the awareness of IGA (e.g., Grigorovici and Constantin, 2004; Schneider and Cornwell, 2005; Acar, 2007; Lee and Faber, 2007; Bardzell et al., 2008; Jeong and Biocca, 2012). These studies mostly analyzed the impact of only one characteristic though (i.e., either ad size or spatial position). The current study investigates the effects of brand prominence by examining how both *ad size* and *spatial position* relate to people’s response to the brand placements, manipulating and combining both characteristics into four different placement types (small-peripheral, small-central, large-peripheral, large-central).

Results demonstrate that there are indeed significant changes in effectiveness between different types of placements, with large-central placements obtaining the highest awareness rates, followed by small-central, large-peripheral, and lastly, small-peripheral placements. Spatial position is the most important placement characteristic, with the central placements obtaining the highest brand recall and brand recognition scores. The effect of ad size is much smaller; large placements are not able to lead to significant differences in brand awareness compared to their smaller counterparts.

Finally, we looked for interaction effects of both game controller and brand prominence on brand awareness. Results indicate that for brand name recognition, the effects of our two manipulations indeed interacted with each other, showing that game controller mainly affects the central placements, while the peripheral placements are not influenced. Moreover, there are significant changes in awareness rates between the different placement types in the symbolic controller condition, but not in the mimetic controller condition. We thus observe floor effects for both playing with the mimetic controller and the awareness rates of the peripheral placements. Since playing the game with the mimetic controller proves to be more difficult, the controls of the game take up the majority of the player’s attentional resources, resulting in all brand placements receiving low attention. Moreover, it seems that it is indeed harder for the peripheral (versus central) placements to attract and keep the player’s attention, leading to their awareness rates remaining low in either condition.

In summary, although the sample of our within-subjects experiment was relatively small (*N* = 31), results already show that kinesthetic involvement is a relevant factor to consider when studying or planning to integrate advertising inside digital games. The findings demonstrate that the nature of a game controller can have a significant effect on the processing of IGA, and that this effect can be partially brought back to players’ perceived control over their actions and movements in-game. However, it would be interesting for future research to examine the impact of kinesthetic even further in experimental studies containing a larger pool of participants with different profiles (e.g., differing levels of prior game expertise), looking at other aspects of kinesthetic involvement, employing different kinds of games, etcetera.

For instance, a person’s sense of kinesthetic involvement might also be dependent on his game expertise: experienced players will often learn the controls of a game more quickly (Calleja, 2011), resulting in a faster automation of control. Since our study mostly included experienced gamers, we did not find a moderating effect of this characteristic. However, it might be interesting for future research to take a closer look at the effect of gamer characteristics on the effectiveness of IGA in general and in combination with the impact of kinesthetic involvement in particular.

Moreover, kinesthetic involvement is not only dependent on the type of game controller that is used to play a game; it also relies on the different modes of in-game control that are possible. In some game environments, in-game control can be brought back to the control over a single entity or avatar, which can be interacted with either from a third-person perspective (giving the player a sense of distance) or from a first-person perspective (giving the player a view of the game world through the eyes of the avatar; Calleja, 2011). In other games, players have control over a number of game-pieces or miniatures, either individually or simultaneously, controlling the miniature world by taking on the role of an external, god-like controller (Calleja, 2011). Further, it is important to mention that some types of games are far more focused on the kinesthetic aspect than others. Games involving intense, fast-paced kinesthetic actions such as racing and shooter games, where the player has to constantly manipulate the controller while following the visual cues shown on the screen, often require extreme levels of attentional resources (Apperley, 2006). In other types of games or game genres, the focus may not lie on fast-paced kinesthetic action but on other components of the game (e.g., puzzle games, strategy games). As such, our results may not apply to all game genres and situations. It is therefore advisable for advertisers to carefully select the type of game in which they want to embed their advertisements (i.e., game genre, game console, game controller).

Finally, the results indicate the relevance of brand prominence, showing that spatial position is a more important variable to consider than ad size. Strategically placing advertisements in the center of the player’s viewpoint may prove to be far more effective than randomly placing large advertisements inside a game environment.

## Conflict of Interest Statement

The authors declare that the research was conducted in the absence of any commercial or financial relationships that could be construed as a potential conflict of interest.

## Footnotes

1 Digital Equipment Corporation (1973).

2 Sony Computer Entertainment Europe (2012).
